# Supplementary material for: Machine Learning Algorithms to Predict the Risk of Rupture of Intracranial Aneurysms: a Systematic Review
Source: Clin Neuroradiol. 2024 Nov 15;35(1):3–16. doi: 10.1007/s00062-024-01474-4 (PMC11832721; doi:10.1007/s00062-024-01474-4)
Supplement: Supplementary file 1 — Search strategy—Title & abstract search up till 9th December 2023. [file 62_2024_1474_MOESM1_ESM.docx]

**Supplementary Material**

Article Title: Machine learning algorithms to predict the risk of rupture of intracranial aneurysms: a systematic review.

Journal Name: Clinical Neuroradiology

**Authors**: Karan Daga^1,2^, Siddharth Agarwal^1^, Zaeem Moti^2^, Matthew BK Lee^3^, Munaib Din^2^, David Wood^1^, Marc Modat^1^, Thomas C Booth^1,4^.

**Affiliations**:

^1^ School of Biomedical Engineering & Imaging Sciences, King’s College London, London UK.

^2^ Guy’s and St. Thomas’ NHS Foundation Trust, London UK.

^3^ University College London Hospital NHS Foundation Trust, London UK.

^4^ Department of Neuroradiology, King’s College Hospital, London UK.

**Corresponding Author:**

**Name**: Dr. Thomas C Booth

**Email**: thomas.booth@kcl.ac.uk

**Supplementary Material**

Search strategy - Title & abstract search up till 9th December 2023.

**Ovid EMBASE: 4079 Results**

| **Number** | **Term** | **Results** |
| --- | --- | --- |
| **1** | brain or head or skull or cerebral or cerebrum or intra?cerebral or cranial or cranium or intra?cranial.mp. | 3392753 |
| **2** | exp Brain/ | 1607042 |
| **3** | aneurysm*.mp. | 240601 |
| **4** | exp Aneurysm/ or exp Intracranial Aneurysm | 174770 |
| **5** | ruptur* or burst* or explod* or pop* or breach* or predict* or stability or stable or unstable.mp. | 7777530 |
| **6** | (1 or 2) and (3 or 4) and 5 | 32904 |
| **7** | AI or (artificial adj1 intelligence) or machine learn* or deep learn* or ((deep or machine) adj1 learn*) or neural network* or ((neural or conv*) adj1 (net* or learn* or model*)) or CNN or RNN or convoluted or convnet or computer?assist* or supervised or unsupervised or (semi adj1 supervised).mp. | 453423 |
| **8** | vector machine* or SVM or ((classification or regression or probability or decision) adj1 tree*) or random forest*.mp. | 106167 |
| **9** | ((deep hybrid or cluster* or bayes* or gauss*) adj3 (learn* or model* or net* or algo*)) or algorithm* or automat* or radiomic*.mp. | 1037758 |
| **10** | exp Algorithms/ or exp Artificial Intelligence/ or exp Machine Learning/ or exp Neural Networks, Computer/ or exp Pattern Recognition, Automated/ | 878686 |
| **11** | ((ensemble or transfer or zero shot or reinforcement or dictionary) adj1 (learn* or model* or net* or algo*)).mp. | 26355 |
| **12** | (PCA or principal component analysis or (k adj1 means) or (nearest adj1  neighbo?r) or KNN or (fuzzy adj3 logi*) or isolation forest or hidden markov model or association rule* or feature bag* or score normali#ation).mp. | 196559 |
| **13** | Regression or cox proportional hazard*.mp. | 1726319 |
| **14** | 7 or 8 or 9 or 10 or 11 or 12 or 13 | 3241858 |
| **15** | 6 and 14 | 4079 |

**Ovid Medline: 2647 Results**

| **Number** | **Term** | **Results** |
| --- | --- | --- |
| **1** | brain or head or skull or cerebral or cerebrum or intra?cerebral or cranial or cranium or intra?cranial.mp. | 2495489 |
| **2** | exp Brain/ | 1342435 |
| **3** | aneurysm*.mp. | 180025 |
| **4** | exp Aneurysm/ or exp Intracranial Aneurysm | 135866 |
| **5** | ruptur* or burst* or explod* or pop* or breach* or predict* or stability or stable or unstable.mp. | 5844648 |
| **6** | (1 or 2) and (3 or 4) and 5 | 22330 |
| **7** | AI or (artificial adj1 intelligence) or machine learn* or deep learn* or ((deep or machine) adj1 learn*) or neural network* or ((neural or conv*) adj1 (net* or learn* or model*)) or CNN or RNN or convoluted or convnet or computer?assist* or supervised or unsupervised or (semi adj1 supervised).mp. | 355788 |
| **8** | vector machine* or SVM or ((classification or regression or probability or decision) adj1 tree*) or random forest*.mp. | 76304 |
| **9** | ((deep hybrid or cluster* or bayes* or gauss*) adj3 (learn* or model* or net* or algo*)) or algorithm* or automat* or radiomic*.mp. | 837714 |
| **10** | exp Algorithms/ or exp Artificial Intelligence/ or exp Machine Learning/ or exp Neural Networks, Computer/ or exp Pattern Recognition, Automated/ | 452221 |
| **11** | ((ensemble or transfer or zero shot or reinforcement or dictionary) adj1 (learn* or model* or net* or algo*)).mp. | 23052 |
| **12** | (PCA or principal component analysis or (k adj1 means) or (nearest adj1  neighbo?r) or KNN or (fuzzy adj3 logi*) or isolation forest or hidden markov model or association rule* or feature bag* or score normali#ation).mp. | 136652 |
| **13** | Regression or cox proportional hazard*.mp. | 1189295 |
| **14** | 7 or 8 or 9 or 10 or 11 or 12 or 13 | 2337610 |
| **15** | 6 and 14 | 2647 |

**Web of Science – 3294 results**

| Number | Term | Results |  |
| --- | --- | --- | --- |
| 1 | ALL=(brain or head or skull or cerebral or cerebrum or intra?cerebral or cranial or cranium or intra?cranial) | 4090366 |  |
| 2 | ALL=(aneurysm*) | 238452 |  |
| 3 | ALL=(ruptur* or burst* or explod* or pop* or breach* or predict* or stability or stable or unstable) | 16024526 |  |
| 4 | #1 AND #2 AND #3 | 24030 |  |
| 5 | TI=(((artificial NEAR/0 intelligence) or ((deep or machine) NEAR/0 learn*))) OR AB=((artificial NEAR/0 intelligence) or ((deep or machine) NEAR/0 learn*)) | 626428 |  |
| 6 | ALL=(algorithm* or automat* or radiomic* or computer assist*) | 5149472 |  |
| 7 | TI = (supervised NEAR/2 (learn* or model* or net* or algo*)) OR AB = (supervised NEAR/2 (learn* or model* or net* or algo*)) | 62528 |  |
| 8 | TI = (unsupervised NEAR/2 (learn* or model* or net* or algo*) ) OR AB= (unsupervised NEAR/2 (learn* or model* or net* or algo*) ) | 33886 |  |
| 9 | TI=(semi supervised NEAR/2 (learn* or model* or net* or algo*) ) OR AB=(semi supervised NEAR/2 (learn* or model* or net* or algo*)) | 15617 |  |
| 10 | TI=(deep hybrid NEAR/2 (learn* or model* or net* or algo*) ) OR AB=(deep hybrid NEAR/2 (learn* or model* or net* or algo*) ) | 16756 |  |
| 11 | TI=(bayes* NEAR/2 (learn* or model* or net* or algo*) ) OR AB=(bayes* NEAR/2 (learn* or model* or net* or algo*) ) | 92394 |  |
| 12 | TI=(cluster* NEAR/2 (learn* or model* or net* or algo*) ) OR AB=(cluster* NEAR/2 (learn* or model* or net* or algo*) ) | 124098 |  |
| 13 | TI=(gauss* NEAR/2 (learn* or model* or net* or algo*) ) OR AB=(gauss* NEAR/2 (learn* or model* or net* or algo*) ) | 57812 |  |
| 14 | TI = (((neural or conv*) NEAR/0 (net* or learn* or model*) ) or CNN or convnet or RNN) OR AB = (((neural or conv*) NEAR/0 (net* or learn* or model*) ) or CNN or convnet or RNN) | 689702 |  |
| 15 | TI=(ensemble NEAR/0 (learn* or model* or net* or algo*) ) OR AB=(ensemble NEAR/0 (learn* or model* or net* or algo*) ) | 24783 |  |
| 16 | TI=(PCA or principal component analysis or (k near/0 means) or (nearest near/0 neighbo$r) or KNN or (fuzzy near/0 logi*) or isolation forest or hidden markov model or association rule* or feature bag* or score normali$ation) OR AB=(PCA or principal component analysis or (k near/0 means) or (nearest near/0 neighbo$r) or KNN or (fuzzy near/0 logi*) or isolation forest or hidden markov model or association rule* or feature bag* or score normali?ation) | 530506 |  |
| 17 | TI=((vector machine or SVM or ((classification or regression or probability or decision) NEAR/0 tree*) or random forest)) OR AB=((vector machine or SVM or ((classification or regression or probability or decision) NEAR/0 tree*) or random forest)) | 291707 |  |
| 18 | TI=(regression or cox proportional hazard*) OR AB=(regression or cox proportional hazard*) | 1980400 |  |
| 19 | #5 OR #6 OR #7 OR #8 OR #9 OR #10 OR #11 OR #12 OR #13 OR #14 OR #15 OR #16 OR #17 or #18 | 8048092 |  |
| 20 | #4 AND #19 | 3294 |  |

**Cochrane – 287 results**

| **Number** | **Term** | **Results** |
| --- | --- | --- |
| **1** | brain or head or skull or cerebral or cerebrum or intracerebral or cranial or cranium or intracranial | 146034 |
| **2** | Aneurysm | 4908 |
| **3** | ruptur* or burst* or explod* or pop* or breach* or predict* or stability or stable or unstable | 379008 |
| **4** | MeSH descriptor: [Artificial Intelligence] explode all trees | 2973 |
| **5** | MeSH descriptor: [Neural Networks, Computer] explode all trees | 543 |
| **6** | MeSH descriptor: [Machine Learning] explode all trees | 938 |
| **7** | MeSH descriptor: [Algorithms] explode all trees | 7212 |
| **8** | (artificial NEAR/2 intelligence) or ((deep or machine) NEAR/2 learn*) | 5166 |
| **9** | algorithm or automat* or radiomic* | 33444 |
| **10** | supervised or unsupervised or (semi NEXT supervised) or (deep hybrid or cluster* or bayes* or gauss*) NEAR/2 (learn* or model* or net* or algo*) | 58395 |
| **11** | ((neural or conv*) NEAR/3 (net* or learn* or model*)) or CNN or convnet or RNN or computer?assist* | 14469 |
| **12** | vector machine or SVM or ((classification or regression or probability or decision) NEAR/2 tree*) or random forest* | 9275 |
| **13** | ((ensemble or transfer or zero shot or reinforcement or dictionary) NEAR/2 (learning or model* or net* or algo*)) | 994 |
| **14** | (PCA or principal component analysis or (k NEXT means) or (nearest NEXT neighbo?r) or KNN or (fuzzy NEAR/3 logi*) or isolation forest or hidden markov model or association rule* or feature bag* or score normali?ation) | 14699 |
| **15** | (regression or cox proportional hazard*) | 87380 |
| **16** | #4 or #5 or #6 or #7 or #8 or #9 or #10 or #11 or #12 or #13 or #14 or #15 | 194930 |
| **17** | #1 and #2 and #3 and #16 | 287 |
